# Nanophthalmos: An Update on the Biological Parameters and Fundus Abnormalities

**DOI:** 10.1155/2021/8853811

**Published:** 2021-03-11

**Authors:** Ning Yang, Liang-Liang Zhao, Jia Liu, Lin-Lin Ma, Jin-Song Zhao

**Affiliations:** Department of Ophthalmology, The Second Hospital of Jilin University, Changchun City, China

## Abstract

Nanophthalmos is a clinical phenotype of simple microphthalmos, in which the anterior and posterior segments of the eyeball do not develop into a normal size without other major ocular or systemic anomalies. Typical clinical manifestations of nanophthalmos include short axial length, thickened sclera, small cornea, shallow anterior chamber, and increased lens-to-eye volume ratio. Currently, there is a lack of recognized diagnostic criteria for nanophthalmos. With the development of eye examination technologies, such as biological measurement and imaging examination, visualization and quantification of the eyeball's shape and structure in nanophthalmos can be realized. New clinical features have been reported, which are of great significance for diagnosing and treating nanophthalmos. This review introduces the related concepts of nanophthalmos and the new developments in its clinical characterization.

## 1. Introduction

Microphthalmos is a developmental ocular disorder [1], characterized by eyeballs with ocular axial length (AL) at least two standard deviations smaller than the average in normal eyes, namely, AL <21 mm [2]. There are few epidemiological data on microphthalmos. Those available found its prevalence to be 0.002–0.017% in the United Kingdom and 0.0009% in China [3].

**Figure 1:**
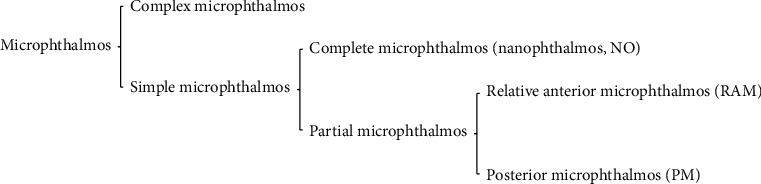
Clinical phenotypes of microphthalmos.

Microphthalmos has many clinical phenotypes (Figure 1), which can occur isolated or in combination with other ocular malformations, and might occur secondary to a systemic syndrome. Microphthalmos is divided into simple and complex types. The former only manifests as a decrease in eyeball volume, without other obvious eye deformities, while the latter occurs alongside other apparent ocular malformations, including chorioretinal coloboma, iris coloboma, and retinal dysplasia [4, 5].

According to the eyeball anterior and posterior segments' length, simple microphthalmos is divided into complete and partial microphthalmos (Table 1).

In complete microphthalmos, also called nanophthalmos, both anterior and posterior segments of the small eyeballs are reduced. Partial microphthalmos is called relative anterior microphthalmos (RAM) when there is a normal posterior segment and a short anterior segment and posterior microphthalmos (PM) when the anterior segment is normal, but the posterior segment is short. It should be noted that RAM also includes eyeballs with reduced anterior segment and normal AL in some documents [6]. Such conditions do not belong to the microphthalmos category.

Nanophthalmos is caused by the stagnation of eyeball development after the embryonic fissure closes [7]. Its characteristic clinical manifestations include short AL, small cornea, shallow anterior chamber, and increased lens-to-eyeball volume ratio [8]. Abnormalities of the choroid and retina can be found in the fundus. Nanophthalmos diagnosis mainly relies on ophthalmological and imaging examinations aimed to obtain the eyeball's biological parameters.

It is important to fully understand and accurately identify its relevant clinical features for accurate nanophthalmos diagnosis and effective treatment. However, previous studies lack a detailed and complete discussion on the clinical characteristics of nanophthalmos. Many clinical features are often overlooked, leading to delays in its diagnosis and treatment. New clinical features of nanophthalmos have been reported as they can now be visually demonstrated with newly developed technologies. This article reviews the classification of microphthalmos, its related concepts, and clinical features to improve ophthalmologists' understanding of the disorder.

## 2. Measured Biological Parameters

### 2.1. Short Ocular Axial Length

The average ocular AL of a normal adult is 22.00–25.00 mm [9]. Duke-Elder [10] was the first to define nanophthalmos and described its AL as 16.00–18.50 mm, but cases that meet this criterion are rare. To date, there is no clear consensus on how to define the AL of nanophthalmos. As a result, there are differences in the inclusion criteria among nanophthalmic studies in terms of AL, which cause confusion when attempting to compare the results in different reports. In most studies, the AL of nanophthalmic eyes is at least two standard deviations smaller than the average AL in the normal population, namely, AL <21.00 mm in adults [8, 11]. In other reports, the nanophthalmic cutoff AL values include <20.50 mm [12–15], <20.00 mm [16–19], <18.00 mm [20], or <17.00 mm [21]. Recent studies have found that there is a significant difference in complications' incidence between eyes with AL <20.00 mm and eyes with AL of 20.00–20.99 mm, the former being probably 15 times higher than the latter [22]. It is suggested that research subjects' inclusion based on different AL standards could significantly impact the research results.

### 2.2. Increased Thickness and Structural Disorders of the Sclera

The scleral thickness in a normal adult is 1.00 mm at the back of the sclera, 0.60 mm at the equator, and 0.30 mm at the thinnest part where the extraocular muscles attach [23]. Brockhurst [24] removed a nanophthalmos scleral flap near the equator and measured its thickness to be 2.00 mm. Pathological examination revealed an irregular distribution of scleral collagen fibers, swelling, and thickness variations [24]. Uyama et al. [25] observed by electron microscopy a large amount of abnormal proteoglycan deposition between the scleral fibers. Tailor et al. [26] believed that the scleral thickening and disordered structure are the causes of nanophthalmos. Their occurrence restricts the eyeball growth and is associated with the occurrence of complications such as uveal effusion and choroidal and retinal detachment [27]. Therefore, it is recommended to include increased scleral thickness into nanophthalmos diagnostic criteria [27]. Clinically, B-scan ultrasonography is used to measure the retinal-choroidal-scleral (RCS) combined thickness. This measurement indirectly reflects the scleral thickness because it is difficult to measure the scleral thickness alone [8, 28]. Wu et al. [8] first included RCS thickness >1.70 mm into the diagnostic criteria of nanophthalmos in their 2004 report. Rajendrababu et al. [29] referred to this standard and reported that the average RCS thickness in 60 nanophthalmic patients included in their study was 1.77∼2.20 mm. Kaewsangthong et al. [30] measured the anterior scleral thickness in a nanophthalmic patient using an ultrasound biomicroscope. They aimed to avoid the influence of uveal leakage and detachment on the RCS measurement results. Their results showed that the scleral thickness at the limbus of this patient was 1.26 mm [30], while the scleral thickness at the limbus of a normal eyeball was 0.53 ± 0.14 mm [31].

### 2.3. Corneal Abnormalities

A small cornea is a characteristic manifestation of nanophthalmos and an important parameter for distinguishing nanophthalmos from PM. Most studies include a corneal diameter <11.00 mm as the diagnostic criteria for nanophthalmos [32, 33]. Relhan et al. [14] found that all nanophthalmos patients included in their study showed a steep cornea, with an average corneal curvature >46 D, while the normal corneal curvature was 43–44 D. Altan et al. [34] found that the cornea of patients with nanophthalmos showed higher biomechanical parameters, including corneal hysteresis and corneal resistance factor. These could lead to excessively high intraocular pressure (IOP) in these patients. Other corneal changes related to nanophthalmos include an irregular cornea, corneal opacity, and corneal vascularization [35–37].

### 2.4. Shallow Anterior Chamber

A shallow anterior chamber (AC) is a characteristic manifestation of nanophthalmos and an important factor in distinguishing nanophthalmos from PM. The AC depth in normal adults is 3.14–3.60 mm [14]. Yalvac et al. [13] reported an average AC depth of 2.30 ± 0.36 mm in nanophthalmos patients, while Zhang et al. [15] reported a depth of 1.38 ± 0.10 mm, which is lower than that in previous reports. Shallow AC increases the risk for secondary angle-closure glaucoma in patients with nanophthalmos and the difficulty of intraocular surgery.

### 2.5. Increased Lens-to-Eyeball Volume Ratio

The lens thickness of normal adults is 4.00–4.45 mm [38], while in patients with nanophthalmos, it is normal or enlarged. Rajendrababu et al. [28] reported an average lens thickness of 4.27 ± 0.70 mm in nanophthalmos eyes. A small eyeball with a normal or enlarged lens volume results in a significant increase in the lens-to-eyeball volume ratio (LEVR). The LEVR of normal adults is 4%, while it is 11–32% in nanophthalmic patients [15, 39]. An increase in LEVR makes nanophthalmic patients prone to secondary angle-closure glaucoma. Patients with nanophthalmos show high hyperopia because the increase in LEVR causes objects to be focused behind the retina [20]. Singh et al. [32] reported that all 32 nanophthalmic patients included in their study had hyperopia with an average diopter of +13.6 D (ranges from +7.25 D to +20.00 D). Jung et al. [12] reported that 88.2% of nanophthalmic patients with cataract needed to be implanted with at least +30 D intraocular lenses, which posed an implantation challenge.

## 3. Morphological Changes of the Nanophthalmic Fundus

### 3.1. Choroidal Changes

The subfoveal choroidal thickness (SFCT) in normal adults is 272–448 *μ*m while it is significantly thicker in patients with nanophthalmos. Demircan et al. [40] first described the SFCT of nanophthalmic patients quantitatively in 2014. They found that the average SFCT in the nanophthalmos group was 551.30 ± 87.00 *μ*m, while it was 330.5 ± 46.0 *μ*m in the control group. These results are consistent with those of Aksoy et al. [41]. Except for the increase in SFCT, patients with nanophthalmos also show a relative increase in the choroidal thickness (CT) on the nasal side. The CT of normal adults is the thickest at the top, followed by the fovea, and then the temporal and bottom sides, with the thinnest part on the nasal side [42, 43]. Unlike in normal eyes, the CT in the nanophthalmos case reported by Kaneko et al. [44] was the thickest at the bottom, followed by the nasal side, with the temporal side being the thinnest part of the choroid. During normal development, the choroid extends from the optic disc to the temporal side, while the nasal side becomes thinner. As the choroid and sclera come from the same source, the choroid of nanophthalmic patients does not develop properly, so it cannot fully stretch. The result is accumulation near the optic disc and an increase in the CT on the nasal side. Besides, Aksoy et al. [41] described the choroidal vasculature in nanophthalmos patients in their 2020 report. They suggested that the choroidal luminal area and total choroid area in nanophthalmic patients increase significantly, but their ratio remains similar to that in the normal group.

### 3.2. Increased Retinal Thickness

The report of Demircan et al. [40] showed that the average central macular thickness (CMT) in nanophthalmic patients (331.90 ± 78.90 *μ*m) was significantly higher than in the control group (268.90 ± 24.30 *μ*m). There is a significant negative correlation between AL and CML [45], which is consistent with the results reported by Bijlsma et al. [45]. In nanophthalmic patients, scleral thickening does not affect the retinal neuroepithelial layer's development but prevents the choroidal and retinal pigment epithelium growth. It is speculated that the increased CMT in nanophthalmos patients is due to the slow choroidal and retinal pigment epithelium growth, which leads to the redundancy of the neural retinal layer [46].

### 3.3. Macular Folds

Nanophthalmos might be accompanied by various macular folds, including papillomacular folds (PMF) and macular radial folds [47]. PMF is most common in PM patients, but the reports of Bijlsma et al. [45] and Liu et al. [48] showed that PMF could also be present in nanophthalmic patients.

The anatomical components of PMF include the thickened ganglion cell layer, inner plexiform layer, inner nuclear layer, outer plexiform layer, and highly concentrated neuroretinal layer. However, as part of the neural retina, the outer membrane and the ellipsoid zone are not involved in PMF formation [45]. It is currently speculated that scleral thickening hinders the development of the choroid and retinal pigment epithelium but does not affect the development of the retinal neuroepithelial layer, leading to the PMF development, similar to the pathogenesis of increased retinal thickness [46].

### 3.4. Absence or Hypoplasia of the Foveal Avascular Zone

The human retina has three vascular plexuses: the radial vascular plexus around the optic papilla, the superficial vascular plexus, and the deep vascular plexus. They are located in the retinal nerve fiber layer, ganglion cell layer, and inner nuclear layer, respectively [49]. The superficial and deep vascular plexuses form a special noncapillary area in the center of the macula. Due to the lack of support from the vascular network structure, the central part of the macula forms a small depression under the action of the mechanical force. This depression is known as the fovea or foveal avascular zone (FAZ) [49, 50]. Walsh and Goldberg [51] reported that the FAZ in eight eyes of four nanophthalmic patients had an abnormal appearance in optical coherence tomography (OCT) and fluorescein angiography. The studies by Funakoshi et al. [52] and Yanik Odabas et al. [53] reported similar findings. A normal FAZ is very important for achieving a central vision of 20/20 or higher [51]. Previous studies have found that even in the absence of known complications, such as angle-closure glaucoma or uveal effusion syndrome, the best-corrected visual acuity of nanophthalmic patients rarely exceeds 20/40 [47]. The exact cause of poor vision in nanophthalmic patients is still unclear, and the lack or underdevelopment of the FAZ might constitute a new explanation for this.

### 3.5. Crowded Optic Disc

Crowded optic disc (pseudo-papillary edema) presents as unclear optic disc borders and bulging optic disc [54]. It is common in the fundus of PM patients and has also been reported in nanophthalmic patients [28]. Tay et al. [55] reported that 14 eyes of 17 nanophthalmic patients had crowded optic discs. The scleral tube of patients with nanophthalmos is small, and the crowded optic disc might be related to the dense arrangement of the optic nerve fibers into the small scleral tube [54].

## 4. Conclusion

Nanophthalmos diagnosis is challenging. As a rare developmental disorder, nanophthalmos often manifests as high hyperopia in its early stage. As this can be corrected by eyeglasses, early recognition and diagnosis are not easy. In addition, the etiology and pathogenesis of nanophthalmos have not been fully elucidated. Nanophthalmos diagnosis depends on meeting specific clinical characteristics. The use of inconsistent criteria for clinical diagnosis in different studies leads to differences in research results. Recent research gives us a reason to reevaluate the basic criteria that define nanophthalmos. We need a grading standard that corresponds to different risks of complications in nanophthalmos. In short, a comprehensive and accurate understanding of the biological parameters and fundus abnormalities in nanophthalmos is crucial for its early identification and diagnosis. It could also help understand the mechanism and suggest treatment directions for nanophthalmos-associated complications.

## Figures and Tables

**Table 1:** Comparison of three clinical phenotypes of simple microphthalmos.

Clinical phenotype	Clinical features
Nanophthalmos	Short axial length caused by shortening of the anterior and posterior segments, accompanied by thickened sclera
Relative anterior microphthalmos	Short axial length caused by shortening of the anterior segment, with a normal-sized posterior segment and without scleral thickening
Posterior microphthalmos	Short axial length caused by shortening of the posterior segment, with a normal-sized anterior segment and thickened sclera
